# Complementary and synergistic therapeutic effects of compounds found in Kampo medicine: analysis of daikenchuto

**DOI:** 10.3389/fphar.2015.00159

**Published:** 2015-08-04

**Authors:** Toru Kono, Mitsuo Shimada, Masahiro Yamamoto, Atushi Kaneko, Yuji Oomiya, Kunitsugu Kubota, Yoshio Kase, Keiko Lee, Yasuhito Uezono

**Affiliations:** ^1^Center for Clinical and Biomedical Research, Sapporo Higashi Tokushukai HospitalSapporo, Hokkaido, Japan; ^2^Pathophysiology and Therapeutics, Faculty of Pharmaceutical Sciences, Hokkaido UniversitySapporo, Japan; ^3^Department of Surgery, Institute of Health Biosciences, Graduate School of Medicine, Tokushima UniversityTokushima, Japan; ^4^Kampo Scientific Strategies Division, Tsumura Research Laboratories, Tsumura & CO.Ami, Japan; ^5^Kampo Scientific Strategies Division, International Pharmaceutical Development Department, Tsumura & CO.Tokyo, Japan; ^6^Division of Cancer Pathophysiology, National Cancer Center Research InstituteTokyo, Japan

**Keywords:** Kampo, traditional Japanese herbal medicine, daikenchuto, adrenomedullin, hydroxy-α-sanshool, transient receptor potential ankyrin 1, potassium leak channels

## Abstract

Herbal medicines have been used in Japan for more than 1500 years and traditional Japanese medicines (Kampo medicines) are now fully integrated into the modern healthcare system. In total, 148 Kampo formulae are officially approved as prescription drugs and covered by the national health insurance system in Japan. However, despite their long track record of clinical use, the multi-targeted, multi-component properties of Kampo medicines, which are fundamentally different from Western medicines, have made it difficult to create a suitable framework for conducting well-designed, large-scale clinical trials. In turn, this has led to misconceptions among western trained physicians concerning the paucity of scientific evidence for the beneficial effects of Kampo medicines. Fortunately, there has been a recent surge in scientifically robust data from basic and clinical studies for some of the Kampo medicines, e.g., daikenchuto (TU-100). Numerous basic and clinical studies on TU-100, including placebo-controlled double-blind studies for various gastrointestinal disorders, and absorption, distribution, metabolism and excretion (ADME) studies, have been conducted or are in the process of being conducted in both Japan and the USA. Clinical studies suggest that TU-100 is beneficial for postoperative complications, especially ileus and abdominal bloating. ADME and basic studies indicate that the effect of TU-100 is a composite of numerous actions mediated by multiple compounds supplied via multiple routes. In addition to known mechanisms of action via enteric/sensory nerve stimulation, novel mechanisms via the TRPA1 channel and two pore domain potassium channels have recently been elucidated. TU-100 compounds target these channels with and without absorption, both before and after metabolic activation by enteric flora, with different timings and possibly with synergism.

## Kampo: History and comparison with western drugs

Japanese traditional herbal medicine, called Kampo, is prescribed for the treatment of a wide array of diseases and conditions, including postoperative ileus, to alleviate the adverse effects of anticancer therapies (such as neurotoxicity, oral mucositis, and anorexia), and to manage the behavioral and psychological symptoms of dementia (Kono et al., 2009; Matsuda et al., 2013; Okada et al., 2015; Shimada et al., 2015). Because Kampo is fully integrated into the modern healthcare system in Japan (Motoo et al., 2011), it is neither a folk remedy nor an alternative therapy. Kampo medicines are dispensed at all the university, national, and foundation hospitals in Japan as prescription drugs, frequently in combination with western drugs. Rooted in Chinese medicine, knowledge of Kampo formulae have been transmitted from generation to generation for 1500 years. However, due to the problem of cultivating and procuring identical species of some of the herbs in the Chinese formulae, together with the limited maritime commerce at the time, Kampo followed a decidedly unique path of development in the Japanese archipelago. Consequently, there are stark differences between Japanese and Chinese herbal formulae.

Until recently, it was much more difficult to ensure the safety of traditional medicines made from natural sources by comparison to modern drugs. However, this preconceived idea has now been overturned by the establishment of a robust quality control system for Kampo prescriptions, which ensures that the cultivation and harvest of botanical raw materials are in accord with World Health Organization Good Agricultural and Collection Practices guidelines for medicinal plants. Moreover, Kampo medicines are now manufactured in accord with both Japanese good manufacturing practice (GMP) and Kampo GMP guidelines, the former provided by law and the latter by self-imposed standards introduced by an association of industrial bodies. In addition, at least for the top selling Kampo products, which comprise over 80% of the market share, extensive component analysis and quality inspection for residual agrichemicals, heavy metals, aflatoxin, microorganisms, and other contaminants at critical steps in the manufacturing process guarantee the manufacture of safe, high-quality, and standardized Kampo products. Since 1967 when the Japanese government initially approved four prescriptions, the number of approved Kampo prescriptions has been steadily increasing, and at present 148 Kampo prescriptions (Japan Pharmaceutical Information Center Kampo extract granules for ethical use 2014) are covered by the national health insurance system and are officially registered by the Japanese Ministry of Health as multicomponent remedies containing extracts derived mainly from plant-based and mineral-based substances. As prescription drugs, Kampo is considered to be in the same class as western drugs. In recent years, the Food and Drug Administration has displayed a keen interest in Kampo, such that daikenchuto (TU-100), the most widely used Kampo in Japan, has been approved as an investigational new drug and multiple clinical trials examining its safety and efficacy are currently underway (www.clinical trails.gov). The first trial conducted at the Mayo Clinic showed that TU-100 significantly accelerated colonic motility in healthy volunteers in direct comparison to placebo (Manabe et al., 2010). Furthermore, investigators at the Universities of Chicago, California, and Oxford have undertaken studies to clarify the pharmacological mechanisms of Kampo in line with their emerging interest in multicomponent remedies (Ueno et al., 2014; Wang et al., 2014).

Nonetheless, there are western-trained physicians in every country who continue to deprecate herbal medicines, owing to a fundamental lack of understanding concerning the differences between Kampo and western drugs (Kono et al., 2009). As described in Table 1, the ideal drug in western medicine is a magic bullet with a single target, which in reality frequently acts on unintended targets producing unwanted side effects. By contrast, Kampo contains multiple components that act synergistically and cooperatively on multiple targets, resulting in a substantially lowered risk of developing side effects (Terasawa, 2004a). Another major difference is that a candidate, novel compound in western medicine can take anywhere from 10 to 15 years to verify its efficacy in pharmacokinetic and basic studies as well as placebo-controlled clinical trials before it is approved as a pharmaceutical drug for the market. By contrast, Kampo has been clinically used in humans and its content refined empirically for approximately 1500 years (Terasawa, 2004b). Given that rigorous scientific investigations of Kampo have only commenced in the last few decades, allopathic physicians have necessarily been skeptical about their use. Fortunately, however, several Kampo products, including daikenchuto, are currently subjected to stringent scientific analyses commensurate with those employed for conventional new drug development.

**Table 1:** **Differences between Western and Kampo medicines**.

		**Ideal**		
**Western (Allopathic) medicines**	**Single compound**		**Single target (Magic bullet)**	**High selectivity No side effect**
		**Reality**		
			**Unintended multiple targets**	**Efficacious but with serious side effects**
Kampo medicines	Compatible compound groups		Intended multiple targets	Efficacious with few side effects

## Daikenchuto

**Figure 1:**
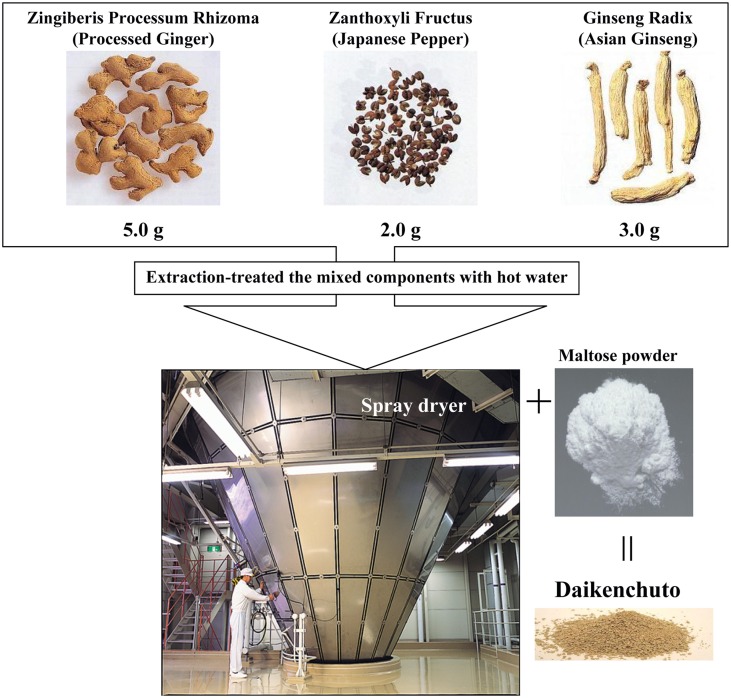
**Daikenchuto components and its manufacturing process**. Daikenchuto is a herbal formulation consisting of processed ginger, Japanese pepper, and ginseng radix. The normal adult dosage is between 7.5 and 15 g given as two or three doses.

Daikenchuto has been officially registered since 1986 and is the most widely prescribed Kampo in Japan with the greatest amount of preclinical and clinical evidence (Kono et al., 2009). The traditional herbal medicine daikenchuto is an extract containing three medicinal herbs, which are also well-known culinary herbs. The three herbs are Japanese pepper (Zanthoxylum fruit), processed ginger (*Zingiberis Siccatum* Rhizoma), and ginseng (*Ginseng radix*). The traditional medicine is made by mixing the herbs in defined ratios, followed by extraction using hot water, and finally the extract is made into a powder (Figure 1). Daikenchuto is primarily administered for the treatment of two symptoms: abdominal bloating and cold sensation in the abdomen. The effect of daikenchuto in alleviating abdominal bloating accompanying postoperative ileus appears to correlate with its effect on improving colonic motility as confirmed in several placebo-controlled, double-blind trials (Kono et al., 2009; Manabe et al., 2010; Iturrino et al., 2013; Okada et al., 2013; Shimada et al., 2015). TU-100 is also known as one of the representative prescriptions for the treatment of chronic constipation (Iizuka and Hamamoto, 2015).

**Figure 2:**
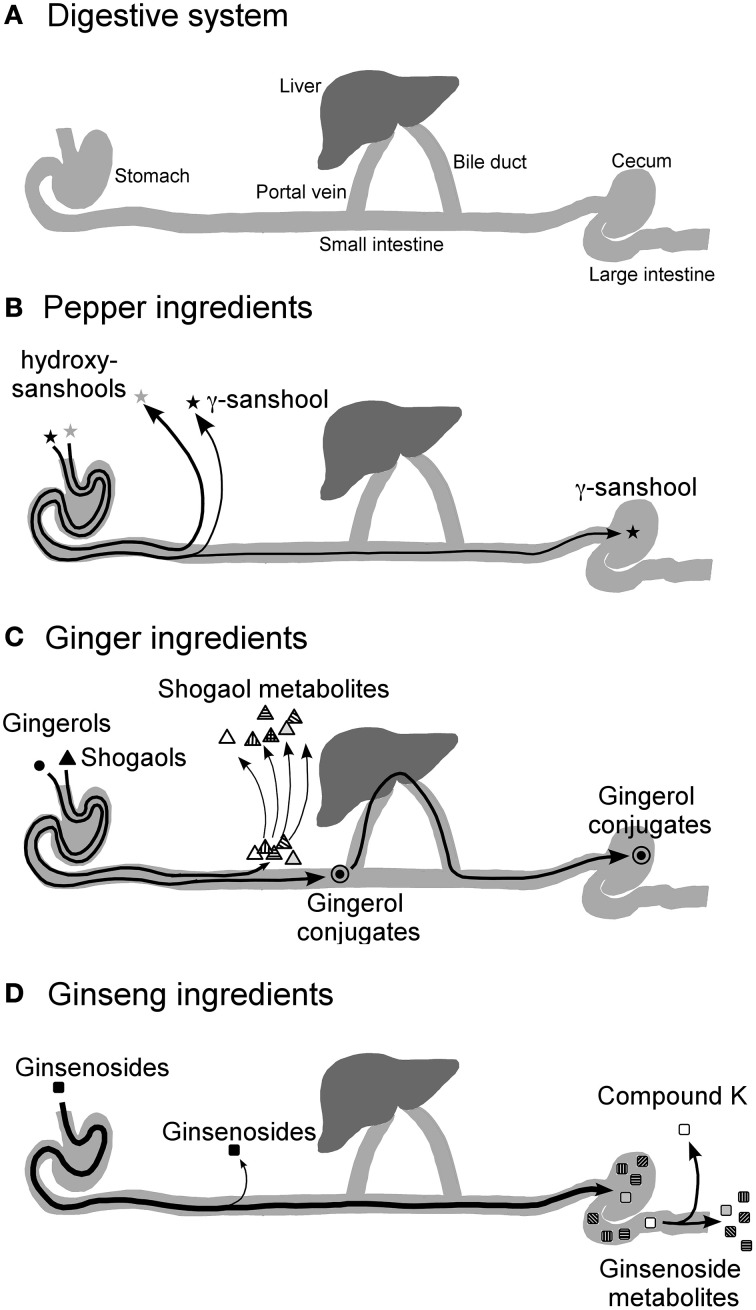
**Diagrammatic representation of the hypothesized journey of TU-100 compounds after ingestion**. The concentrations of TU-100 compounds and their conjugates after oral administration of TU-100 in rat intestinal, portal blood and peripheral blood and in human ileal effluent and peripheral blood were measured in several PK studies (Munekage et al., 2011, 2013; Watanabe et al., in press). The results are summarized as indicated in the figure. The diagram of digestive sysytem **(A)**, and the fate of Japanese pepper **(B)**, Ginger **(C)** and ginseng **(D)** ingredients are shown. The effect of TU-100 on the intestines should be a composite of multiple actions by multiple compounds supplied via multiple routes. Quoted from Watanabe et al. (in press).

Meanwhile, mechanistic studies are increasingly demonstrating that the prokinetic effect of daikenchuto could be ascribed to its effect on neurotransmitters such as acetylcholine and calcitonin gene-related peptide (CGRP) (Nagano et al., 1999; Shibata et al., 1999; Jin et al., 2001). However, until recently, compound analysis and pharmacokinetic studies, which are considered indispensable in conventional drug development, have been sparse because of difficulties in simultaneously tracking multiple components in the Kampo medicine. Nonetheless, the advent of technologies such as liquid chromatography-mass spectrometry for the identification of compounds and characterization of their kinetics with greater accuracy and precision (Munekage et al., 2011, 2013) has facilitated the analysis of daikenchuto. The use of 3D-HPLC has helped identify the multiple compounds in Kampo, but the pharmacokinetic data has revealed for the first time that these compounds are absorbed and metabolized at different rates. For instance, our data showed that the main active compounds of Japanese pepper, namely, hydroxy-α-sanshool (HAS) and hydroxy-β-sanshool (HBS), produce transient peaks, indicating their rapid absorption at high concentrations (HAS in the order of μM) as well as their rapid elimination over a period of a few hours (Munekage et al., 2011, 2013). The main active compounds of processed ginger ([6]-shogaol and [6]-gingerol) are also absorbed, but then rapidly metabolized and conjugated after absorption. Consequently, the plasma concentrations of [6]-shogaol and [6]-gingerol are < 1% compared with that of the Japanese pepper compounds (Watanabe et al., in press). By contrast, most of the ginseng compounds remain unabsorbed. Moreover, pharmacokinetic analysis of luminal content after administration revealed that Japanese pepper compounds are rapidly absorbed before reaching the colonic lumen (Watanabe et al., in press). Processed ginger compounds are transformed into various metabolites in the upper small intestine and liver with some compounds entering the enterohepatic circulation (Watanabe et al., in press). Ginseng compounds reach the colon intact and are absorbed only after they are metabolized by the enteric microbiota (Watanabe et al., in press). Among the bacterial metabolites of ginseng compounds, some have been found to have antitumor and anti-inflammatory properties such as compound K (20-O-(β-D-glucopyranosyl)-20(S)-protopanaxadiol) (Wakabayashi et al., 1997; Bae et al., 2002; Tachikawa and Kudo, 2004). Taken together, one of the key findings from the pharmacokinetic study is that the compounds are absorbed at different rates and act accordingly on various target cells (Figure 2). The significance of this finding will be elaborated after the explanation of one of the other important pharmacological effects of daikenchuto.

**Figure 3:**
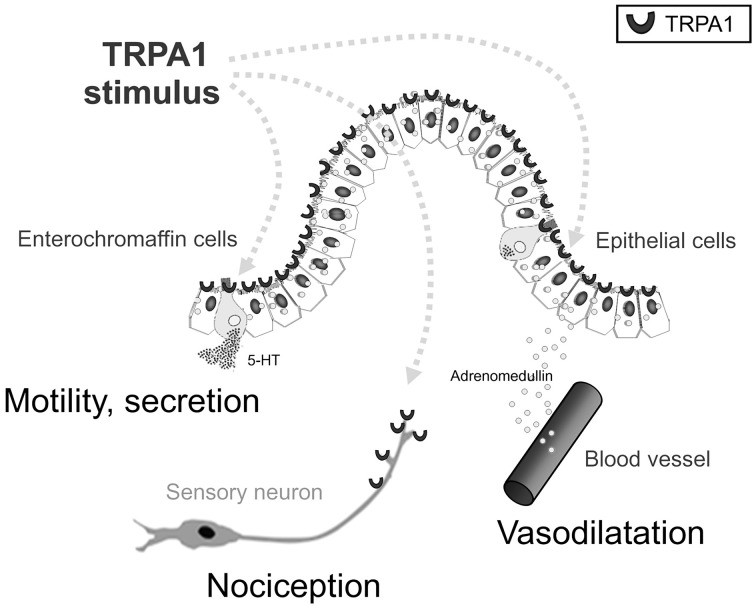
**Multi-target actions of intraluminal TRPA1 agonists**. Gut TRPA1 elicits physiological and pathophysiological responses by three distinct mechanisms. TRPA1 activators, in this case shogaols, gingerols and hydroxysanhools, have three potential target cells: intestinal epithelial (IE) cells, enterochromaffin (EC) cells, and TRPA1-positive sensory neurons. As a result of TRPA1 stimulation, TRPA1 agonists stimulate IE cells to release ADM, EC cells to release 5-HT, and sensory neurons to release CGRP/substance P, respectively, resulting in physiological and biodefensive responses in vasodilatation, motility, secretion, and pain signaling. Quoted from Kono et al. (2013).

As mentioned earlier, daikenchuto is primarily prescribed for the treatment of abdominal bloating and cold sensation in the abdomen. The clinical improvement in relieving cold sensation in the abdomen after administration of daikenchuto has previously been attributed to its effect in improving intestinal blood flow (Takayama et al., 2010). We have found that the critical players responsible for these effects are the two peptides, CGRP and adrenomedullin (ADM) (Kono et al., 2008, 2009, 2010, 2011, 2013; Kaneko et al., 2013). Although the two peptides have strikingly similar vasodilatory, anti-inflammatory, and antibacterial properties, they are produced by different tissues, i.e., CGRP by neuronal tissues and ADM by a wide range of various tissues other than neuronal cells, especially by endothelial and vascular smooth muscle cells in heart, lung, kidney, and cerebral vasculature (Brain and Grant, 2004). The intestinal epithelial cells (IECs) are particularly endowed with the ability to produce ADM because we have found that daikenchuto administered into the intestinal lumen causes an immediate increase in blood flow in both the small and large intestines (Kono et al., 2011, 2013). When this data is presented at academic meetings in the West, one of the frequently asked questions is whether the effect of daikenchuto is equivalent to that induced by capsaicin. We have discovered that this is not the case because the daikenchuto-induced increase in blood flow is not abrogated by the inhibitors of transient receptor potential vanilloid receptor 1 (TRPV1), a capsaicin receptor. Another common point of contention is whether an augmentation of blood flow will aggravate pre-existing inflammation. Our results from the experimental colitis model have shown that blood flow increases in areas of poor circulation, while it remains unchanged in regions of high levels of inflammation (Kono et al., 2011). This is most likely due to the mobilization and exhaustion of the two primary targets of daikenchuto, i.e., CGRP and ADM, in inflamed areas and that the Kampo remedy itself is neither CGRP nor ADM. In addition, mechanistic probing of ADM production has revealed that daikenchuto induces ADM release from the IECs in a dose- and time-dependent manner largely via its active compound, [6]-shogaol. Furthermore, [6]-shogaol appears to stimulate a type of Ca^2+^channel called the transient receptor potential ankyrin 1 (TRPA1), to promote ADM release from the IECs as well (Kono et al., 2013). It should be noted that TRPA1 is expressed in enterochromaffin cells and sensory neurons as well as in IEC. HAS, [6]-gingerol and [6]-shogaol are all known to have TRPA1-stimulating activity (Riera et al., 2009). Because the compounds are transported to various target cells via different routes as described in the previous paragraph, TU-100 could stimulate TRPA1 in all these cell types (Figure 3). CGRP-mediated effects on intestinal blood flow (Murata et al., 2002) and substance P-mediated contraction of intestinal smooth muscle (Satoh et al., 2001) by TU-100 is thought to occur via sensory nerves. The possible involvement of TRPA1-induced serotonin release from enterochromaffin cells in the TU-100-mediated prokinetic effect is currently being investigated in our laboratory.

**Figure 4:**
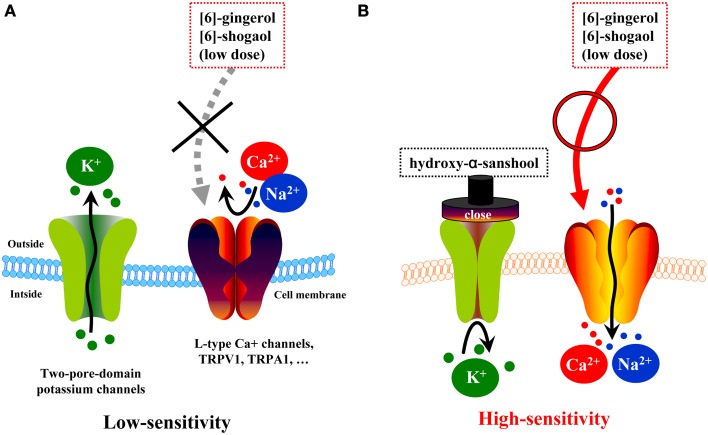
**Hypotheses on synergism of TU-100 compounds on colonic motility. (A)** Two-pore-domain potassium channels (i.e., KCNKs family channels) are expressed in many types of excitable cells throughout the body and have been implicated in various cellular functions, including the maintenance of the resting potential and regulation of excitability. Low doses of ginger compounds cannot evoke action potentials. **(B)** One of compounds of daikenchuto, hydroxy-α-sanshool, acts as a blocker of two-pore-domain potassium leak channels (KCNK3 and KCNK9) and alters the excitability of a cell via voltage-activated cation channels. Low doses of ginger compounds can evoke action potentials.

However, pharmacokinetic data has shown that the plasma concentration of [6]-shogaol is low and does not correspond with the effective dose observed in *in vitro* studies, but such a dose would likely be considered high in the lumen. It has been confirmed that daikenchuto administered directly using a long tube is clinically effective in patients (Yasunaga et al., 2011). In subsequent studies, we analyzed the kinetics of Japanese pepper compounds, HAS and HBS, which are known agonists of TRPA1 and TRPV1 receptors (actions resembling those of processed ginger compounds) and antagonists of potassium leak channels, two-pore-domain subfamily K (KCNK) (Kubota et al., 2015). KCNK channels exist in cell membranes of excitatory cells such as neurons and muscles as highly regulated, K^+^-selective leak channels (Mackinnon et al., 1998; Bautista et al., 2008). As such, these channels are fundamental to maintaining the resting potential of the cell and regulating cellular excitability. In the neurons, KCNK channels regulate the opening of voltage-gated Na+ channels, which generate action potentials. Recent studies have shown that HAS and HBS accelerate colonic motility by inhibiting KCNK3 and KCNK9 channels in the intestinal smooth muscle and neuronal cells (Kubota et al., 2015). In light of these findings, combined with the pharmacokinetic data, we postulated the hypothesis outline in Figure 4. Daikenchuto administration causes an initial blockade of KCNK channels in the intestinal smooth muscle and neuronal cells by the action of the Japanese pepper compounds, which leads to increased membrane sensitivity. Thus, there is a decreased threshold for additional exogenous stimuli, such as subsequent exposure to ginger and ginseng compounds. In short, these results suggest that daikenchuto compounds could induce a therapeutic effect at concentrations lower than those required for each compound to exert its effect alone. Indeed, preliminary investigations, using experimental systems described above, suggest that Japanese pepper and processed ginger, and Japanese pepper and ginseng, exhibit synergistic effects on intestinal blood flow and colonic motility, respectively (unpublished data). Unraveling the precise mechanisms underpinning these synergistic effects will be an enormous task. Nevertheless, taking into consideration all the available data, our hypothesis appears to be both robust and credible. We believe that systems biology will be particularly useful for elucidating multicomponent remedies and has the potential of producing groundbreaking results that could instigate a paradigm shift in healthcare.

Contemporary Kampo is a classic example of the harmonization between traditional herbal medicine and modern medicine. Rigorous scientific investigations are now beginning to reveal the complex therapeutic effects mediated by Kampo. The ancient adage of maximizing the temporal differences in pharmacological effects may be similar to the modern concept of a combination chemotherapy regimen, except that one Kampo prescription by itself fulfills the role of a combination regimen. We conjecture that Kampo is likely to benefit many patients, particularly those whose condition remains recalcitrant to conventional treatments. As such, we advocate the worldwide availability of Kampo medicines.

## Author contributions

TK, MS, MY, and YU conceived the experiment. TK, MY, AK, KK, and YO conducted the experiment and analyzed the data. TK and KL wrote the paper. YK contributed ideas and improved the manuscript.

### Conflict of interest statement

Masahiro Yamamoto, Yoshio Kase, Keiko Lee, Atushi Kaneko, Yuji Oomiya, and Kunitsugu Kubota are employed by Tsumura & Co. Toru Kono, Mitsuo Shimada, and Yasuhito Uezono have financial interests from Tsumura & Co. relevant to this research.

## Abbreviations

GMP: good manufacturing practice
TU-100: daikenchuto
CGRP: calcitonin gene-related peptide
HAS: hydroxy-α-sanshool
HBS: hydroxy-β-sanshool
ADM: adrenomedullin
IECs: intestinal epithelial cells
TRPV1: transient receptor potential vanilloid receptor 1
TRPA1: transient receptor potential ankyrin 1
KCNK: potassium leak channels, two-pore-domain subfamily K.

## References

[B1] Bae E. A. Choo M. K. Park E. K. Park S. Y. Shin H. Y. Kim D. H. (2002). Metabolism of ginsenoside R(c) by human intestinal bacteria and its related antiallergic activity. Biol. Pharm. Bull. 25, 743–747. 10.1248/bpb.25.743 12081140

[B2] Bautista D. M. Sigal Y. M. Milstein A. D. Garrison J. L. Zorn J. A. Tsuruda P. R.. (2008). Pungent agents from Szechuan peppers excite sensory neurons by inhibiting two-pore potassium channels. Nat. Neurosci. 11, 772–779. 10.1038/nn.2143 18568022 PMC3072296

[B3] Brain S. D. Grant A. D. (2004). Vascular actions of calcitonin gene-related peptide and adrenomedullin. Physiol. Rev. 84, 903–934. 10.1152/physrev.00037.2003 15269340

[B4] Iizuka N. Hamamoto Y. (2015). Constipation and Herbal medicine. Front. Pharmacol. 6:73. 10.3389/fphar.2015.00073 25904866 PMC4389350

[B5] Iturrino J. Camilleri M. Wong B. S. Linker Nord S. J. Burton D. Zinsmeister A. R. (2013). Randomised clinical trial: the effects of daikenchuto, TU-100, on gastrointestinal and colonic transit, anorectal and bowel function in female patients with functional constipation. Aliment. Pharmacol. Ther. 37, 776–785. 10.1111/apt.12264 23451764

[B6] Jin X. L. Shibata C. Naito H. Ueno T. Funayama Y. Fukushima K.. (2001). Intraduodenal and intrajejunal administration of the herbal medicine, dai-kenchu-tou, stimulates small intestinal motility via cholinergic receptors in conscious dogs. Dig. Dis. Sci. 46, 1171–1176. 10.1023/A:1010690624187 11414290

[B7] Kaneko A. Kono T. Miura N. Tsuchiya N. Yamamoto M. (2013). Preventive effect of TU-100 on a type-2 model of colitis in mice: possible involvement of enhancing adrenomedullin in intestinal epithelial cells. Gastroenterol. Res. Pract. 2013, 384057. 10.1155/2013/384057 24348533 PMC3852085

[B8] Kono T. Kaneko A. Hira Y. Suzuki T. Chisato N. Ohtake N.. (2010). Anti-colitis and -adhesion effects of daikenchuto via endogenous adrenomedullin enhancement in Crohn's disease mouse model. J. Crohns Colitis 4, 161–170. 10.1016/j.crohns.2009.09.006 21122500

[B9] Kono T. Kaneko A. Omiya Y. Ohbuchi K. Ohno N. Yamamoto M. (2013). Epithelial transient receptor potential ankyrin 1 (TRPA1)-dependent adrenomedullin upregulates blood flow in rat small intestine. Am. J. Physiol. Gastrointest. Liver Physiol. 304, G428–G436. 10.1152/ajpgi.00356.2012 23275609 PMC3566615

[B10] Kono T. Koseki T. Chiba S. Ebisawa Y. Chisato N. Iwamoto J.. (2008). Colonic vascular conductance increased by Daikenchuto via calcitonin gene-related peptide and receptor-activity modifying protein 1. J. Surg. Res. 150, 78–84. 10.1016/j.jss.2008.02.057 18561951

[B11] Kono T. Mishima H. Shimada M. Morita S. Sakamoto J. (2009). Preventive effect of goshajinkigan on peripheral neurotoxicity of FOLFOX therapy: a placebo-controlled double-blind randomized phase II study (the GONE Study). Jpn. J. Clin. Oncol. 39, 847–849. 10.1093/jjco/hyp100 19734172

[B12] Kono T. Omiya Y. Hira Y. Kaneko A. Chiba S. Suzuki T.. (2011). Daikenchuto (TU-100) ameliorates colon microvascular dysfunction via endogenous adrenomedullin in Crohn's disease rat model. J. Gastroenterol. 46, 1187–1196. 10.1007/s00535-011-0438-2 21808981

[B13] Kubota K. Ohtake N. Ohbuchi K. Mase A. Imamura S. Sudo Y.. (2015). Hydroxy-alpha sanshool induces colonic motor activity in rat proximal colon: a possible involvement of KCNK9. Am. J. Physiol. Gastrointest. Liver Physiol. 308, G579–G590. 10.1152/ajpgi.00114.2014 25634809 PMC4385894

[B14] Mackinnon R. Cohen S. L. Kuo A. Lee A. Chait B. T. (1998). Structural conservation in prokaryotic and eukaryotic potassium channels. Science 280, 106–109. 10.1126/science.280.5360.106 9525854

[B15] Manabe N. Camilleri M. Rao A. Wong B. S. Burton D. Busciglio I.. (2010). Effect of daikenchuto (TU-100) on gastrointestinal and colonic transit in humans. Am. J. Physiol. Gastrointest. Liver Physiol. 298, G970–975. 10.1152/ajpgi.00043.2010 20378829

[B16] Matsuda Y. Kishi T. Shibayama H. Iwata N. (2013). Yokukansan in the treatment of behavioral and psychological symptoms of dementia: a systematic review and meta-analysis of randomized controlled trials. Hum. Psychopharmacol. 28, 80–86. 10.1002/hup.2286 23359469

[B17] Motoo Y. Seki T. Tsutani K. (2011). Traditional Japanese medicine, Kampo: its history and current status. Chin. J. Integr. Med. 17, 85–87. 10.1007/s11655-011-0653-y 21390572

[B18] Munekage M. Ichikawa K. Kitagawa H. Ishihara K. Uehara H. Watanabe J.. (2013). Population pharmacokinetic analysis of daikenchuto, a traditional Japanese medicine (Kampo) in Japanese and US health volunteers. Drug Metab. Dispos. 41, 1256–1263. 10.1124/dmd.112.050112 23545807

[B19] Munekage M. Kitagawa H. Ichikawa K. Watanabe J. Aoki K. Kono T.. (2011). Pharmacokinetics of daikenchuto, a traditional Japanese medicine (kampo) after single oral administration to healthy Japanese volunteers. Drug Metab. Dispos. 39, 1784–1788. 10.1124/dmd.111.040097 21724872

[B20] Murata P. Kase Y. Ishige A. Sasaki H. Kurosawa S. Nakamura T. (2002). The herbal medicine Dai-kenchu-to and one of its active components [6]-shogaol increase intestinal blood flow in rats. Life Sci. 70, 2061–2070. 10.1016/S0024-3205(01)01552-1 12148698

[B21] Nagano T. Itoh H. Takeyama M. (1999). Effect of Dai-kenchu-to on levels of 3 brain-gut peptides (motilin, gastrin and somatostatin) in human plasma. Biol. Pharm. Bull. 22, 1131–1133. 10.1248/bpb.22.1131 10549871

[B22] Okada K. Kawai M. Hirono S. Miyazawa M. Shimizu A. Kitahata Y.. (2015). Perioperative administration of Daikenchuto (TJ-100) reduces the postoperative paralytic ileus in patients with pancreaticoduodenectomy. Hepatogastroenterology 62, 466–471. 25916084

[B23] Okada K. Kawai M. Uesaka K. Kodera Y. Nagano H. Murakami Y.. (2013). Effect of daikenchuto (TJ-100) on postoperative bowel motility and on prevention of paralytic ileus after pancreaticoduodenectomy: a multicenter, randomized, placebo-controlled phase II trial (The JAPAN-PD study). Jpn. J. Clin. Oncol. 43, 436–438. 10.1093/jjco/hyt005 23365113

[B24] Riera C. E. Menozzi-Smarrito C. Affolter M. Michlig S. Munari C. Robert F.. (2009). Compounds from Sichuan and Melegueta peppers activate, covalently and non-covalently, TRPA1 and TRPV1 channels. Br. J. Pharmacol. 157, 1398–1409. 10.1111/j.1476-5381.2009.00307.x 19594761 PMC2765304

[B25] Satoh K. Hashimoto K. Hayakawa T. Ishige A. Kaneko M. Ogihara S.. (2001). Mechanism of atropine-resistant contraction induced by Dai-kenchu-to in guinea pig ileum. Jpn. J. Pharmacol. 86, 32–37. 10.1254/jjp.86.32 11430470

[B26] Shibata C. Sasaki I. Naito H. Ueno T. Matsuno S. (1999). The herbal medicine Dai-Kenchu-Tou stimulates upper gut motility through cholinergic and 5-hydroxytryptamine 3 receptors in conscious dogs. Surgery 126, 918–924. 10.1016/S0039-6060(99)70033-4 10568192

[B27] Shimada M. Morine Y. Nagano H. Hatano E. Kaiho T. Miyazaki M.. (2015). Effect of TU-100, a traditional Japanese medicine, administered after hepatic resection in patients with liver cancer: a multi-center, phase III trial (JFMC40-1001). Int. J. Clin. Oncol. 20, 95–104. 10.1007/s10147-014-0678-2 24595550

[B28] Tachikawa E. Kudo K. (2004). Proof of the mysterious efficacy of ginseng: basic and clinical trials: suppression of adrenal medullary function *in vitro* by ginseng. J. Pharmacol. Sci. 95, 140–144. 10.1254/jphs.FMJ04001X2 15215636

[B29] Takayama S. Seki T. Watanabe M. Takashima S. Sugita N. Konno S.. (2010). The effect of warming of the abdomen and of herbal medicine on superior mesenteric artery blood flow - a pilot study. Forsch. Komplementmed. 17, 195–201. 10.1159/000317845 20829597

[B30] Terasawa K. (2004a). Evidence-based reconstruction of Kampo medicine: part-III-How should kampo be evaluated? Evid. Based Complement. Alternat. Med. 1, 219–222. 10.1093/ecam/neh046 15841254 PMC538517

[B31] Terasawa K. (2004b). Evidence-based reconstruction of Kampo medicine: part I-Is Kampo CAM? Evid. Based Complement. Alternat. Med. 1, 11–16. 10.1093/ecam/neh003 15257321 PMC442105

[B32] Ueno N. Hasebe T. Kaneko A. Yamamoto M. Fujiya M. Kohgo Y.. (2014). TU-100 (Daikenchuto) and ginger ameliorate anti-CD3 antibody induced T cell-mediated murine enteritis: microbe-independent effects involving Akt and NF-kappaB suppression. PLoS ONE 9:e97456. 10.1371/journal.pone.0097456 24857966 PMC4032249

[B33] Wakabayashi C. Hasegawa H. Murata J. Saiki I. (1997). *In vivo* antimetastatic action of ginseng protopanaxadiol saponins is based on their intestinal bacterial metabolites after oral administration. Oncol. Res. 9, 411–417. 9436194

[B34] Wang L. Mogami S. Karasawa H. Yamada C. Yakabi S. Yakabi K.. (2014). Preventive effect of rikkunshito on gastric motor function inhibited by L-dopa in rats. Peptides 55, 136–144. 10.1016/j.peptides.2014.02.011 24631952 PMC5944319

[B35] Watanabe J. Kaifuchi N. Kushida H. Matsumoto T. Fukutake M. Nishiyama M. (in press). Intestinal, portal peripheral profiles of daikenchuto (TU-100)'s active ingredients after oral administration. Pharmacol. Res. Perspect. 10.1002/prp2.165 PMC4618637 26516578

[B36] Yasunaga H. Miyata H. Horiguchi H. Kuwabara K. Hashimoto H. Matsuda S. (2011). Effect of the Japanese herbal kampo medicine dai-kenchu-to on postoperative adhesive small bowel obstruction requiring long-tube decompression: a propensity score analysis. Evid. Based Complement. Alternat. Med. 2011:264289. 10.1155/2011/264289 21584269 PMC3092181

